# Post-harvest chitosan treatment suppresses oxidative stress by regulating reactive oxygen species metabolism in wounded apples

**DOI:** 10.3389/fpls.2022.959762

**Published:** 2022-08-02

**Authors:** Sabina Ackah, Yang Bi, Sulin Xue, Salimata Yakubu, Ye Han, Yuanyuan Zong, Richard Atinpoore Atuna, Dov Prusky

**Affiliations:** ^1^College of Food Science and Engineering, Gansu Agricultural University, Lanzhou, China; ^2^Department of Food Science and Technology, Shanghai Ocean University, Shanghai, China; ^3^Department of Food Science and Technology, University for Development Studies, Tamale, Ghana; ^4^Department of Post-harvest Science of Fresh Produce, Agricultural Research Organization, Rishon LeZion, Israel

**Keywords:** apple fruit, wound, chitosan, reactive oxygen species, antioxidant enzymes, ascorbate-glutathione cycle

## Abstract

Mechanical wound on fruit triggers the formation of reactive oxygen species (ROS) that weaken cell walls, resulting in post-harvest losses. This mechanism can be controlled by using fruit preservatives to stimulate fruit antioxidant enzyme activities for the detoxification of ROS. Chitosan is a safe and environmentally friendly preservative that modulates ROS in whole fruits and plant cells, but the effects of chitosan on the ROS metabolism of mechanically wounded apples during storage are unknown. Our study focused on exploring the effects of post-harvest chitosan treatment on ROS production, cell membrane integrity, and enzymatic and non-enzymatic antioxidant systems at fruit wounds during storage. Apple fruits (cv. Fuji) were artificially wounded, treated with 2.5% (w/v) chitosan, and stored at room temperature (21–25°C, RH = 81–85%) for 7 days. Non-wounded apples were used as healthy controls. The results showed that chitosan treatment stimulated the activities of NADPH oxidase and superoxide dismutase and increased the formation of superoxide anions and hydrogen peroxide in fruit wounds. However, malondialdehyde, lipoxygenase, and membrane permeability, which are direct biomarkers to evaluate lipid peroxidation and membrane integrity, were significantly decreased in the wounded fruits after chitosan treatment compared to the wounded control fruits. Antioxidant enzymes, such as peroxidase and catalase activities, were induced by chitosan at fruit wounds. In addition, ascorbate-glutathione cycle-related enzymes; ascorbate peroxide, monodehydroascorbate reductase, dehydroascorbate reductase, and glutathione reductase and the content of substrates, mainly ascorbic acid, dehydroascorbate, reduced glutathione, and glutathione, were increased at fruit wounds by chitosan compared to the wounded control fruits. Our results show that wounding stimulated the production of ROS or oxidative stress. However, treatment with chitosan triggered antioxidant systems to scavenge ROS and prevent loss of fruit membrane integrity. Therefore, chitosan promises to be a favorable preservative in inducing tolerance to stress and maintaining fruit quality.

## Introduction

Apples (*Malus domestica Borkh* cv. Fuji) are temperate fruits commonly produced worldwide (Wang et al., 2018), and are significant sources of minerals, vitamins, polyphenols, anthocyanins, and organic acids with lots of health benefits (Feliciano et al., 2010). Nevertheless, apples are prone to mechanical wounding during harvesting and handling, which serves as a means for pathogen invasion leading to loss of water and disease infection (Ackah et al., 2022). Wounding may also cause loss of cell membrane integrity leading to spoilage (Adiletta et al., 2019; Gong et al., 2019).

Reactive oxygen species (ROS) metabolism contributes to the strengthening of fruit cell walls, which begins with an oxygen burst for ROS production to induce defense (Han et al., 2018; Xue et al., 2019). ROS metabolism and phenylpropanoid metabolism can lead to the formation of lignin and suberin to promote fruit wound healing (Ackah et al., 2022). Hydroxyl radical (OH), superoxide anion (O${}_{2}^{-}$), hydrogen peroxide (H_2_O_2_), and singlet oxygen (^1^O_2_) are examples of ROS produced in cellular organelles due to biological activities like photosynthesis and respiration (Adiletta et al., 2021), and are largely formed due to oxidative stress (Fooladi vanda et al., 2019). Mechanical wounding is a major abiotic stress that stimulates ROS production in fruit wounds (Ackah et al., 2022). O${}_{2}^{-}$ and H_2_O_2_ generation in response to wounding has been observed in apples (Shao et al., 2010; Zhang et al., 2020) and potatoes (Rui et al., 2020; Yang et al., 2020; Zhu et al., 2021). Also, the increase of H_2_O_2_ in tomatoes (Lu et al., 2021), oranges (Zeng et al., 2010), apples, and pineapples (Wu et al., 2013) due to wounding stress has been reported. The immediate outburst of ROS at the early stage of wounding is a signal to induce tolerance to stress. However, excess ROS can cause lipid peroxidation leading to membrane damage and the oxidation of proteins, DNA, and carbohydrate (Adiletta et al., 2021). The production of ROS due to mechanical damage accelerates senescence, contributing to the downgrading and post-harvest loss of fresh horticultural produce, which is recorded to be about 51% post-harvest loss (Lu et al., 2021). Therefore, efficient and safe treatment like the use of plant elicitors is critical for maintaining fruit quality since the chemical control method poses health issues.

Chitosan is a hydroxylated polysaccharide derivative of chitin obtained from the outer shell of crustaceans (Duan et al., 2019). Chitosan is a non-toxic, environmentally friendly edible polymer with antifungal characteristics that can trigger plant defense responses (Romanazzi et al., 2017). Chitosan coating controls *Colletotrichum gloeosporioides, Botrytis cinerea, Penicillium digitatum* and *Penicillium italicum, etc*. in many horticultural crops (Wang and Gao, 2013; Zhang et al., 2015; Zhao et al., 2018; Obianom et al., 2019; Peian et al., 2021; Ackah et al., 2022).

Research shows that chitosan regulates ROS production by enhancing signal transduction pathways to induce defense (Xiangchun et al., 2012), especially H_2_O_2_ signal molecules (Lin et al., 2005). In previous studies, chitosan increased antioxidant enzyme activities to decrease ROS in loquat fruit (Lin et al., 2020). Chitosan stimulates antioxidant activities to remove excess ROS to maintain membrane integrity in strawberries, pears, and grapes (Li et al., 2010a; Wang and Gao, 2013; Petriccione et al., 2015). Also, the membrane integrity of apple and fig is maintained when coated with chitosan by the eliciting of antioxidant enzyme activities (Adiletta et al., 2019; Zhao et al., 2021). Furthermore, chitosan treatment elicits antioxidant enzymes activities to reduce malondialdehyde (MDA) in maize and wheat plants during growth to induce tolerance to salt stress (Geng et al., 2020; Sadak and Talaat, 2021), and drought stress in maize plants and white clover (Li et al., 2017; Almeida et al., 2020).

Many research works have proven that chitosan can regulate ROS metabolism and induce defense in whole and healthy fruits during storage and plant cells. However, knowledge about the ability of chitosan to regulate the formation and elimination of ROS in wounded apples has not been reported. Thus, this study ought to examine the impact of post-harvest chitosan treatment on (1) superoxide anion (O${}_{2}^{-}$) and hydrogen peroxide (H_2_O_2_) accumulation, (2) fruit's membrane integrity, and (3) antioxidant and ascorbate-glutathione cycle-related enzymes and product and substrate content in apples under wounding stress during storage.

## Materials and methods

### Fruit and chitosan treatment

Apples (*Malus domestica Borkh*. cv. Fuji) were harvested from the Tiaoshan farm in Jingtai, Gansu, China. Apples without injury, dirt, and of similar size were selected and carefully packed into perforated paper boxes. The fruits were subsequently brought to the laboratory for the experiment.

Chitosan of ≥ 90% deacetylation, was purchased from WN Group of Publishers Ltd., France, as a chemical treatment. The preliminary test results determined the chitosan concentration used. In a preliminary test, the wounded apples were treated with chitosan at 1, 1.5, 2, 2.5, and 3%, respectively. It was found that 2.5% (w/v) chitosan was significantly (*p* ≤ 0.01) effective in inducing resistance against *Penicillium expansum*, reducing weight loss, and promoting H_2_O_2_ and lignin accumulation in fruit wounds. Thus, 2.5% chitosan solution was chosen as our treatment in this study, which was prepared by dissolving a 25 g of chitosan chemical agent in 1,000 ml distilled water at room temperature.

### Apple fruit wounding and treatment

The Zhang et al. (2020) method was followed in the fruit wounding and treatment process. To disinfect the surfaces of apples, they were washed with 0.1% sodium hypochlorite for 3 min, then rinsed with distilled water, and dried at room temperature for 3 h. The fruits were further sterilized using 75% alcohol. Three artificial wounds (circle 7.3–7.8 mm radius and 1 mm depth) were inflicted on the equatorial region of each fruit with a sterile scalpel (Deli, NO. 2034, China). A group of wounded and unwounded fruits was completely soaked in 2.5% chitosan solution for 10 min, while another group of wounded and non-wounded fruits was treated with distilled water as control. The wounded and non-wounded fruits were packed in aerated polyethylene bags and stored in the dark at room temperature (21–25°C, relative humidity = 81–85%) for 7 d. Two independent experiments with three replicates were performed in a completely randomized design. The treatments were (i) wounded fruit treated with chitosan (W + chitosan), (ii) wounded fruit treated with distilled water (W + water), (iii) non-wound fruit treated with chitosan (N + chitosan), and (iv) non-wounded fruit treated with distilled water (N + water).

### Determination of cell membrane permeability

Xue et al. (2020) method was used to determine the cell membrane permeability of wounded fruit. A total of 10 g fruit sample was kept in 40 ml of distilled water and a laboratory conductivity meter (DDS-307A; RIDAO, Shanghai) was used to measure fruit conductivity and recorded as E0. The sample was then stored at ambient temperature for 3 h and the conductivity was measured again as E1. The samples in the test tubes were placed in boiling water for 30 min, quickly cooled, and the conductivity was determined and recorded as E2. The fruit's cell membrane permeability was calculated using the formula: Cell membrane permeability (%) = (E1 - E0)/E2 × 100%.

### Sampling

Tissues from the fruit-wounded area were collected according to Zhang et al. (2020) method after 0, 1, 3, 5, and 7 days of wounding. The sample tissues were carefully cut out from the outmost tissue of the wounded surface using a sterilized sharp blade and frozen in nitrogen (liquid). Tissues from non-wounded fruits were collected respectively. The fruit tissues were ground into powder and kept in a freezer (−80°C) for further analysis.

### Assay of reactive oxygen species

Superoxide anion (O${}_{2}^{-}$) and hydrogen peroxide (H_2_O_2_) contents were determined using assay kits supplied by Beijing Solarbio Science and Technology Co. Ltd. The manufacturer's protocols was followed to determine O${}_{2}^{-}$ and H_2_O_2_ contents and results were expressed in μmol g^−1^ FW and mmol g^−1^ FW, respectively.

### Determination of malondialdehyde content and lipoxygenase activity

Malondialdehyde (MDA) content and Lipoxygenase (LOX) activity were determined using MDA and LOX test kits purchased from Beijing Solarbio Science and Technology Co. Ltd. Reagents were used as instructed. MDA and LOX activity results were expressed as nmol g^−1^ and U g^−1^ FW, respectively.

### Determination of some enzymatic activities

Assay kits for the activity of nicotinamide adenine dinucleotide phosphate (NADPH) oxidases (NOX), sodium dismutase (SOD), catalase (CAT), and peroxidase (POD) from Beijing Solarbio Science and Technology Co. Ltd. were used to test the activities of each of the above enzymes. The reagents were added according to the manufacturer's instructions. The results were expressed as U g^−1^ FW for all parameters.

The method described by Han et al. (2021) was employed to extract crude enzymes to determine ascorbate peroxidase (APX), monodehydroascorbate reductase (MDHAR), dehydroascorbate reductase (DHAR), and glutathione reductase (GR) activities. A total of 3 ml of phosphate buffer (100 mM) with pH 7.5 containing 1 mM EDTA was added to 3 g of frozen tissues and centrifuged (4 °C, 8,000 × g) for 25 min. The supernatant collected was used as the crude enzyme. Briefly, the APX activity was determined as described by Han et al. (2021). A total of 2 ml of phosphate buffer (100 Mm, pH 7.5, 1 mM EDTA), 0.8 ml 3 mM ascorbic acid, and 0.5 ml 0.5 mM H_2_O_2_ was added to 200 μl crude enzyme solution. The reaction system was measured at 290 nm and recorded at an interval of 30 s within 2 min. The APX activity was expressed as U g^−1^ FW, U = 0.01 OD_290_ min^−1^.

The MDHAR activity was determined according to the method of Han et al. (2021). A 2 ml phosphate buffer solution (40 mM, pH 8.0), 0.2 ml 10 mM sodium ascorbate, 0.1 ml 40 μM copper sulfate solution, and 0.2 ml of 0.2 mM NADPH were added to 0.5 ml enzyme solution. The reaction system was measured at 340 nm and recorded at an interval of 30 s within 2 min. The MDHAR activity was expressed as U g^−1^ FW, U = 0.01 OD_340_ min^−1^.

DHAR was determined as described by Han et al. (2021). A 2 ml phosphate buffer solution (40 mM, pH 8.0), 300 μl 0.1 mM EDTA-Na_2_, 400 μl 2 mM GSH, and 400 μl 0.5 mM DHA were mixed well with 100 μl crude enzyme solution. The reaction system was measured at 290 nm and recorded at an interval of 30 s within 2 min. The DHAR activity was expressed as U g^−1^ FW, U = 0.01 OD_290_ min^−1^.

The GR activity was measured using the method of Han et al. (2021). A 3 ml phosphate buffer (100 Mm), 0.1 ml 5 mM oxidized glutathione, 30 μl 3 mM NADPH, and 0.2 ml crude enzyme solution were mixed thoroughly. The reaction system was measured at 340 nm and recorded at an interval of 30 s within 2 min. The GR activity was expressed as U g^−1^ FW, U = 0.01 OD_340_ min^−1^.

### Determination of AsA-GSH cycle products contents

Test kits from Beijing Solarbio Science and Technology Co. Ltd. were used to assay the contents of ascorbic acid (AsA), dehydroascorbate (DHA), reduced glutathione (GSH), and glutathione (GSSG). All substrate and product content mentioned were assayed according to the manufacturer's protocol. The AsA, DHA, GSH, and GSSG contents were expressed as nmol g^−1^ FW, μmoL g^−1^ FW, μmol g^−1^ FW, and nmol g^−1^ FW, respectively.

### Statistical analysis

All parameters analyzed were replicated thrice. The averages and standard errors (± SE) of data were calculated using Excel 2016. A one-way analysis of variance (ANOVA) in SPSS 20 (SPSS Inc., United States) was used to determine the effect of the treatments on the response variables. Duncan's multiple range test was used to determine significant differences at *p* < 0.05.

## Results

### Chitosan treatment enhanced O${}_{2}^{\text{–}}$ and H_2_O_2_ production

**Figure 1:**
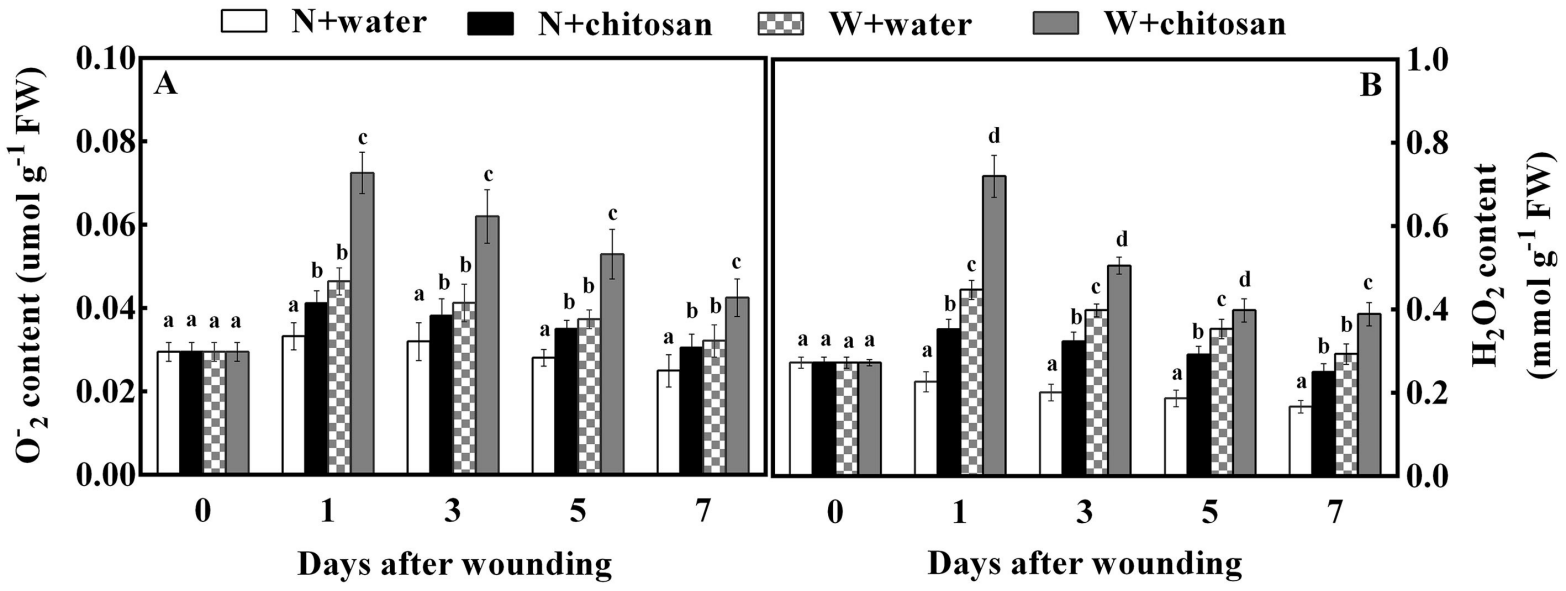
Effect of chitosan treatment on O${}_{2}^{-}$**(A)** and H_2_O_2_ **(B)** content at apple wounds. Non-wounded fruit treated with sterile water (N + water), non-wounded fruit treated with chitosan (N + chitosan), wounded fruit treated with sterile water (W + water), and wounded fruit treated with chitosan (W + chitosan). Vertical bars indicate standard error of means. For each day after wounding, alphabets indicate significant differences (*P* < 0.05).

The generation of O${}_{2}^{-}$ and H_2_O_2_ by ROS is due to oxygen bursts in cells. O${}_{2}^{-}$ and H_2_O_2_ contents in all fruits increased on day 1 and decreased gradually afterward. Throughout the storage days, there were significant differences (*p* < 0.05) between the wounded and non-wounded fruits at each sampling time. In particular, the wounded fruits recorded higher content than non-wounded fruits (Figure 1) due to wounding stress, which triggered ROS formation (O${}_{2}^{-}$ and H_2_O_2_) during storage. Furthermore, the wounded fruits treated with chitosan exhibited a higher content throughout the storage days (Figures 1A,B). The O${}_{2}^{-}$ and H_2_O_2_ content for chitosan-treated wounded fruits were 56% and 32.7% more than the wounded control fruits on days 1 and 7, respectively. These results indicate that chitosan can influence ROS production at fruit wound sites.

### Chitosan treatment reduced cell membrane permeability, MDA content, and LOX activity

**Figure 2:**
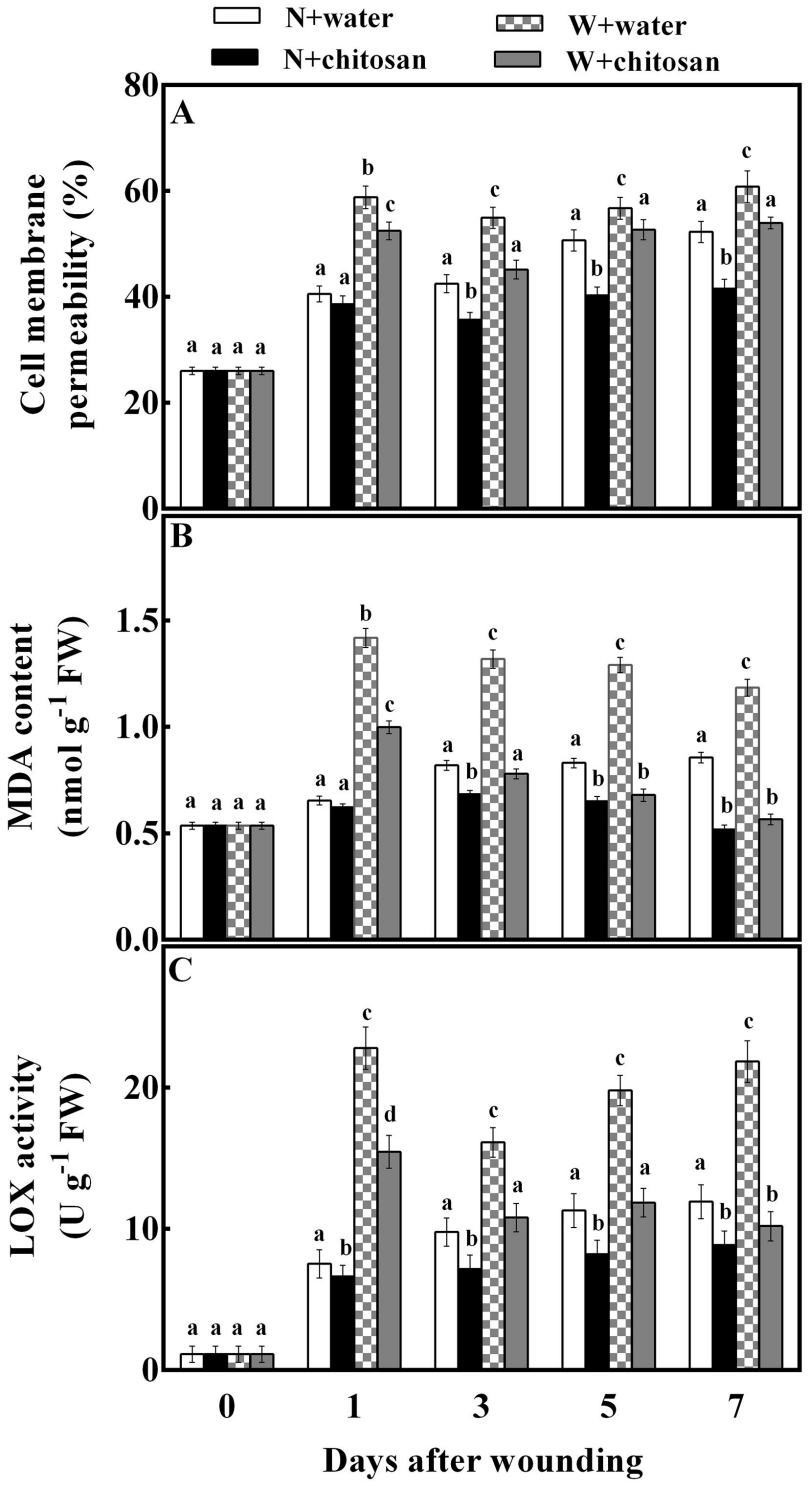
Effect of chitosan treatment on cell membrane permeability **(A)**, MDA content **(B)**, and LOX activity at apple wounds **(C)**. Non-wounded fruit treated with sterile water (N + water), non-wounded fruit treated with chitosan (N + chitosan), wounded fruit treated with sterile water (W + water), and wounded fruit treated with chitosan (W + chitosan). Vertical bars indicate standard error of means. For each day after wounding, alphabets indicate significant differences (*P* < 0.05).

Cell membrane permeability, MDA content, and LOX activity are indexes to analyze the rate of membrane integrity loss and lipid oxidation in fruits. In this study, the cell membrane permeability of all wounded fruits increased on day 1, declined on day 3, and increased steadily from day 5 to day 7. In contrast, for non-wounded fruits, it increased as the storage days increased. On each sampling day, the chitosan-treated fruits recorded lower values than the control for wounded and non-wounded fruit. The chitosan-treated wounded fruit was significantly lower (18%) compared to the wounded control fruit on day 3 (Figure 2A) and 26.5% significantly (*p* < 0.05) lower than its non-wounded fruit. The MDA contents peaked on day 1 for both controls and treated wounded fruits. On subsequent days, the contents in control samples reduced slowly while in chitosan-treated fruit reduced sharply. In addition, the MDA content increased in non-wounded fruits with time except on day 7, where the chitosan-treated fruit decreased slightly (Figure 2B). The wounded fruits had comparatively higher MDA content than the non-wounded fruits. However, the chitosan-treated wounded fruits were reduced by 52.2% on day 7 compared to the wounded control fruits (Figure 2B). The LOX activity of all wounded fruits increased on day 1, declined on day 3, and increased continuously toward the end of storage. At the same time, the LOX activity of the non-wounded fruits increased with storage days. The wounding stress affected the membrane integrity by causing a higher LOX activity in the wounded fruits than in the non-wounded fruits showing significant differences (*p* < 0.05) in each sampling time (Figure 2). The displayed results also showed that chitosan caused a decrease in the LOX activity in all treated fruits than the controls throughout storage. The chitosan-treated wounded fruits recorded a lower value of 53% compared to the wounded control fruit on day 7 (Figure 2C). These results indicate that chitosan effectively reduced ROS in fruit wounds.

### Chitosan treatment activated NOX, SOD, CAT, and POD enzyme activities

**Figure 3:**
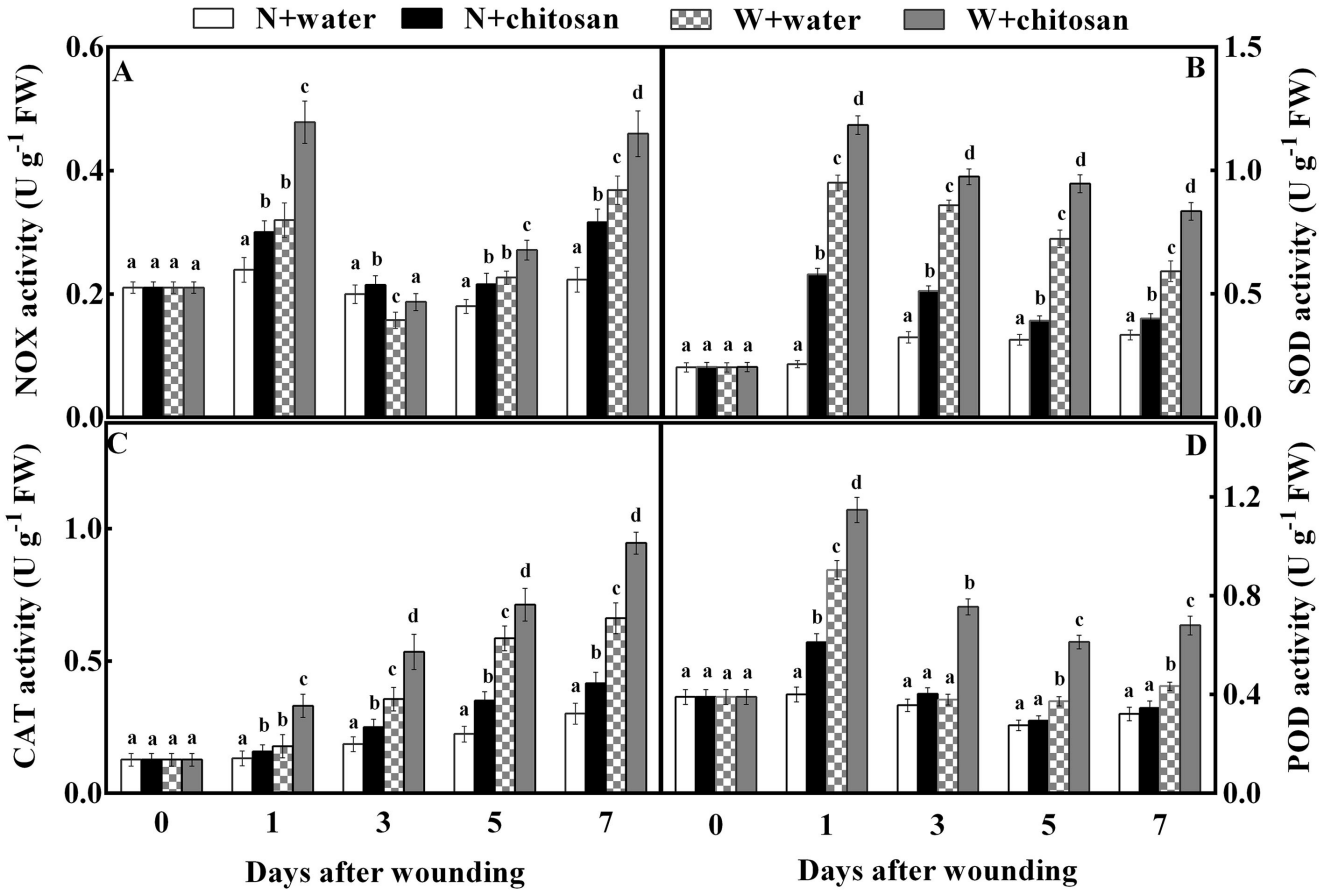
Effect of chitosan treatment on the activities of NOX **(A)**, SOD **(B)**, CAT **(C)**, and POD **(D**). Non-wounded fruit treated with sterile water (N + water), non-wounded fruit treated with chitosan (N + chitosan), wounded fruit treated with sterile water (W + water), and wounded fruit treated with chitosan (W + chitosan). Vertical bars indicate standard error of means. For each day after wounding, alphabets indicate significant differences (*P* < 0.05).

The NOX enzymes are vital enzymes that participate in ROS production in cells. The NOX activity increased on day 1, declined on days 3 and 5, but increased on day 7 for both wounded and non-wounded fruits. From the graph, the wounded fruits had higher NOX activities than the non-wounded fruits showing significant differences (*p* < 0.05) between wounded and their corresponding non-wounded fruits on each storage day. This is attributed to the wounding stress to stimulate ROS production. Consequently, chitosan-treated fruits showed higher activities than control fruits. For instance, chitosan-treated wounded fruit was 50% more than the wounded control fruit on day 1 (Figure 3A).

The presence of antioxidants during storage determines fruits' ability to scavenge excessive ROS to reduce membrane damage. The SOD activity of all fruits peaked on day 1 and decreased gradually toward the end of storage, but the process was much slower in non-wounded fruits as shown in Figure 3B. The chitosan-treated wounded fruit recorded higher SOD activity of 41% than the wounded control fruit on day 7 (Figure 3B). The CAT activity of both wounded and non-wounded fruits increased as the storage time increased, with chitosan-treated fruits having higher activity than the controls. Essentially, the chitosan wounded fruit was 86% more than the wounded control on day 1 (Figure 3C). The POD activity increased on day 1, declined on days 3 and 5, and again increased on day 7 for both wounded and non-wounded fruits. However, the chitosan-treated fruits had higher POD activity than the control fruits throughout the storage days. The POD activity of chitosan-treated wounded fruit was increased by 99% compared to the wounded control fruit on day 3. The results showed significant differences (*p* < 0.05) between wounded and non-wounded fruits at each storage time, where wounded fruits exhibited higher antioxidant enzyme activities compared to their corresponding non-wounded fruits due to wounding stress (Figure 3). However, in each case, chitosan treatment caused an increase in antioxidant enzyme activity, especially in wounded fruits than the control-wounded fruits. Our results showed that chitosan treatment increased antioxidant enzymes at fruit wound sites.

### Chitosan treatment increased AsA-GSH cycle enzyme activities

**Figure 4:**
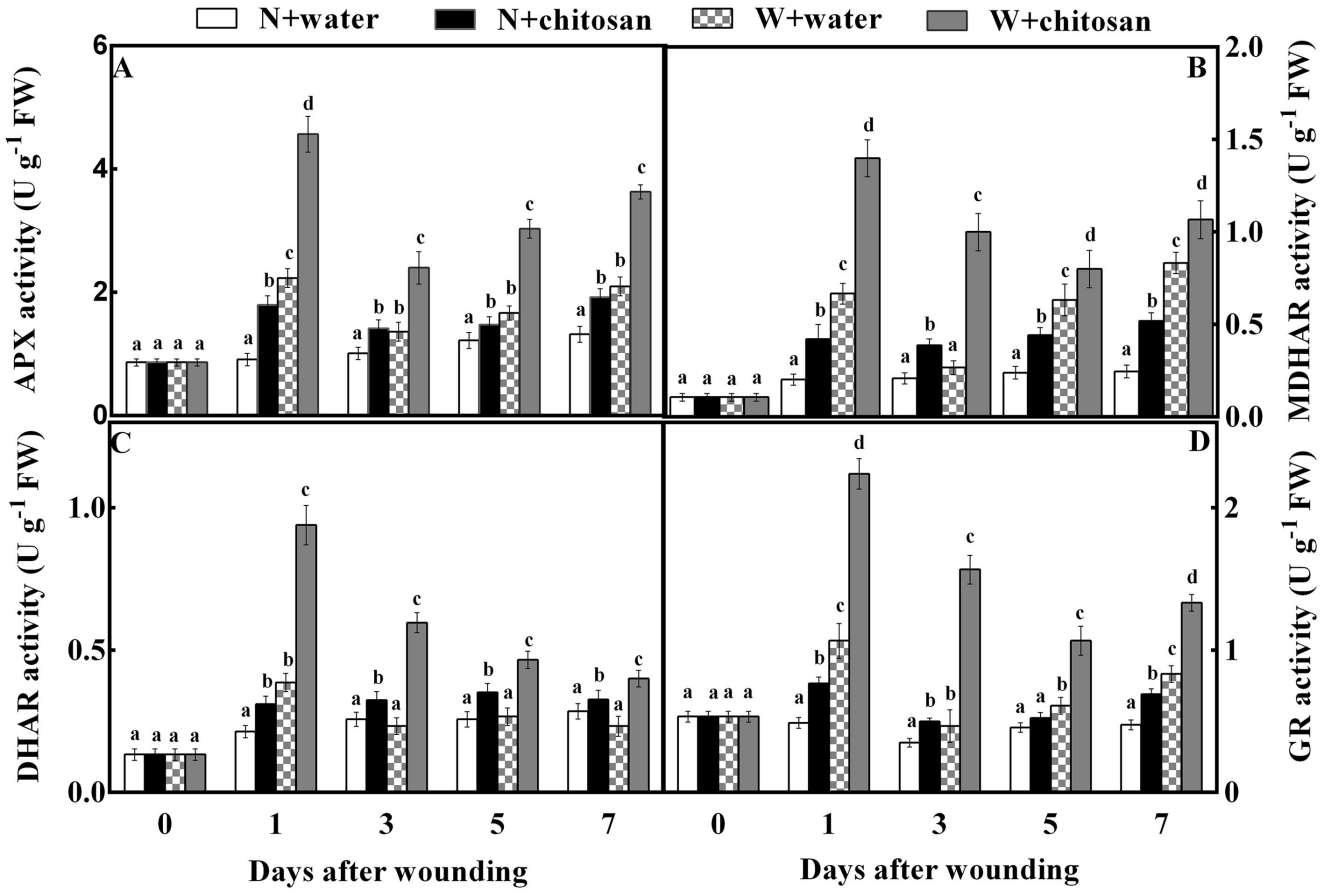
Effect of chitosan treatment on the activities of APX **(A)**, MDHAR **(B)**, DHAR **(C)**, and GR **(D)** at apple wounds. Non-wounded fruit treated with sterile water (N + water), non-wounded fruit treated with chitosan (N + chitosan), wounded fruit treated with sterile water (W + water), and wounded fruit treated with chitosan (W + chitosan). Vertical bars indicate standard error of means. For each day after wounding, alphabets indicate significant differences (*P* < 0.05).

The APX, MDHAR, DHAR, and GR are antioxidant enzymes that aid in ROS scavenging. The APX activity of all fruits increased sharply on day 1, declined on day 3, and again increased continuously on the rest of the healing days. In response to wound stress, the APX activity was higher in wounded fruit than in non-wounded fruit, with significant differences (*p* < 0.05) on each sampling day. The chitosan-treated fruit had higher activities than the control, especially the chitosan-treated wounded fruit (Figure 4A). The chitosan-treated wounded fruit was 1.5-fold higher than the wounded control fruit on day 1 (Figure 4A). The MDHAR and GR activities of chitosan-treated and control-wounded fruit increased sharply on the first day, decreased on days 3 and 5, and again increased on day 7 but increased as storage time increased in non-wounded fruits. Compared to non-wounded fruits, the MDHAR and GR activities of wounded fruit were significantly (*p* < 0.05) higher than in non-wounded fruit due to wounding stress. However, the chitosan-treated fruit recorded higher activities than their control at each sampling time. From the results, the MDHAR- and GR-activity-chitosan-treated wounded fruits were 2.7- and 2.3-folds more than the control-wounded fruits on day 3, respectively. The DHAR activity for all wounded fruits increased on day 1 and decreased slowly from day 3 to day 7.

Meanwhile, in non-wounded fruit, DHAR increased slightly on day 1 and remained steady till the end of storage. Wound stress affected wounded fruits to increase DHAR activity more than non-wounded fruits. Also, chitosan-treated fruits had high activities than control fruits (Figure 4C). The DHAR activity of chitosan-treated wounded fruit increased by 1.5-fold on day 3 compared to the control-wounded fruit (Figure 4C). These results indicate that chitosan elicits antioxidant enzyme activities in the AsA-GSH pathway at the fruit wound sites.

### Chitosan treatment stimulated the synthesis of ascorbate-glutathione cycle products and substrates content

**Figure 5:**
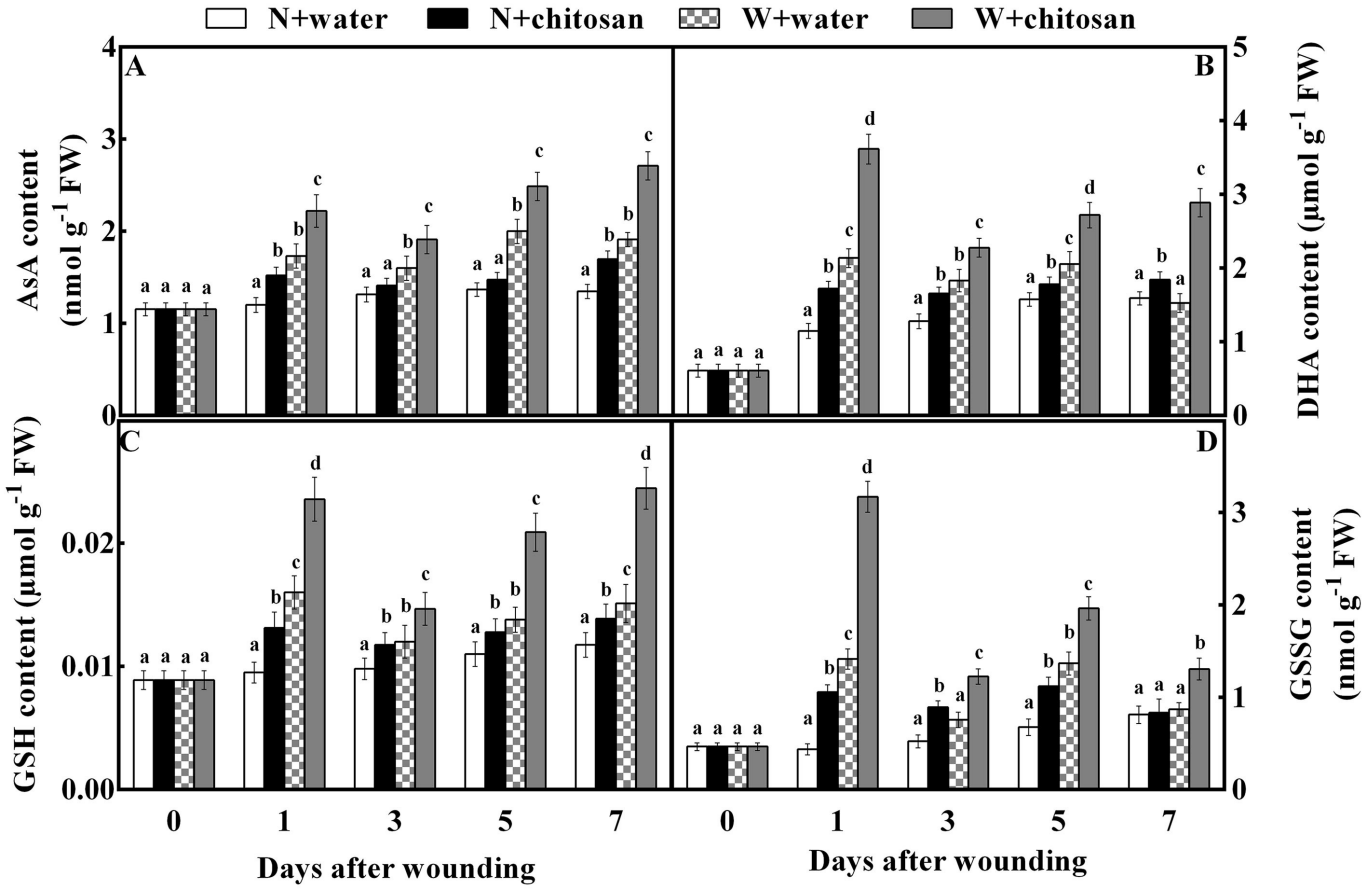
Effect of chitosan treatment on the contents of AsA **(A)**, DHA **(B)**, GSH **(C)**, and GSSG **(D)** at apple wounds. Non-wounded fruit treated with sterile water (N + water), non-wounded fruit treated with chitosan (N + chitosan), wounded fruit treated with sterile water (W + water), and wounded fruit treated with chitosan (W + chitosan). Vertical bars indicate standard error of means. For each day after wounding, alphabets indicate significant differences (*P* < 0.05).

AsA, DHA, GSH, and GSSG (non-enzymatic antioxidants) stimulate enzymatic activity in the AsA-GSH cycle. The AsA content increased continuously throughout the storage time for all fruits as chitosan-treated fruits showed higher activities than their control at each storage time (Figure 5A). The chitosan-treated wounded fruits recorded lower values (42%) than the control-wounded fruits on day 7 (Figure 5B). The DHA and GSH content peaked in all wounded fruits on day 1, decreased on day 3, and again increased on days 5 and 7. In non-wounded fruits, the DHA and GSH activity increased with time. However, chitosan-treated fruit had higher content than the control in each group. The DHA and GSH content of chitosan-treated wounded fruit was more than the control-wounded fruit by 89 and 62% on day 7, respectively (Figures 5B,C). As shown in Figure 5D, the GSSG content in all wounded fruits peaked, declined on day 3, increased on day 5, and again decreased on day 7. The GSSG in non-wounded fruits increased continuously except on day 7, which decreased slightly. Nevertheless, the chitosan-treated wounded fruit had higher content throughout storage, which was 1.2-fold more than the control-wounded fruit on day 1 (Figure 5D). Based on Figure 5, the wound stress stimulated the formation of the antioxidant to induce tolerance than in non-wounded fruits. In addition, chitosan treatment caused an increase in substrates than control, especially in chitosan-treated wounded fruits. These results show that chitosan promotes the production of non-enzymatic antioxidants substrate and product content in the AsA-GSH pathway at the fruit wound sites.

## Discussion

Reactive oxygen species (ROS) are generated from mechanisms in cellular organelles and are highly produced due to stress, including wounding (Adiletta et al., 2021). Research reveals that wounding stimulates H_2_O_2_ production in apples (Shao et al., 2010; Zhang et al., 2020), oranges (Zeng et al., 2010), and tomatoes (Lu et al., 2021). This phenomenon was observed in this study at the early stage of healing, where there was a high content of O${}_{2}^{-}$ and H_2_O_2_ in all wounded fruits than in non-wounded fruits. However, the chitosan treatment stimulated higher O${}_{2}^{-}$ and H_2_O_2_ content in wounded fruit than in the control-wounded fruit (Figure 1). Similarly, chitosan enhanced the production of H_2_O_2_ in fig and pear fruits during storage (Li et al., 2010a; Adiletta et al., 2019). Chitosan acts as a plant elicitor, causing oxygen bursts for $O_{2}^{-}$ and H_2_O_2_ generation at the fruit wound site (Fooladi vanda et al., 2019; Ackah et al., 2022). The immediate outburst of ROS at the early stage of wounding provided the signals to induce stress tolerance. Notwithstanding this effect, the untreated wounded fruits showed lower ROS accumulation than the treated wounded fruits. The O_2_ produced is converted to O${}_{2}^{-}$ with the aid of NADPH oxidase and immediately generates into H_2_O_2_ under the enzymatic action of SOD (Zhang et al., 2020). According to Xue et al. (2020), the enhancement of NOX and SOD activity in the plasma membrane is stimulated by wound stress, leading to the rapid formation of ROS. Similarly, the NOX and SOD activities in wounded fruit were higher than in non-wounded fruit. Our findings revealed that chitosan treatment activated NOX and SOD activity in wounded fruits more than in wounded control fruits (Figure 3). According to Hidangmayum et al. (2019), treating fruit with chitosan can increase the concentration of intracellular Ca^2+^, leading to the activation of calcium-dependent protein kinase (CDPK), which in turn, phosphorylates NOX. Therefore, we believe that chitosan activates the NOX pathway in the wound of fruits or plants by regulating the influx of Ca^2+^ and promotes the production of H_2_O_2_. H_2_O_2_ is also a signal molecule that activates the expression of defense protein by activating the redox-dependent transcription factors (Yang et al., 2020).

Excessive production of ROS in fruits can damage cell membranes (Petriccione et al., 2015; Lin et al., 2020). The lipoxygenase (LOX) activity depicts the deoxygenation of polyunsaturated fatty acids into toxic hydroperoxy fatty acids by ROS (Petriccione et al., 2015). Cell membrane permeability and MDA are the parameters to evaluate the loss of membrane integrity and lipid peroxidation (Lin et al., 2020). Our results show that wounding causes a loss of fruit membrane integrity compared to non-wounded fruits. However, chitosan caused lower content of MDA, cell membrane permeability, and LOX activity at the fruit wound site maintaining membrane integrity than the control-wounded fruit (Figure 2). This is because of increased antioxidant activity by chitosan to scavenge excess ROS at fruit wounds. Similarly, chitosan significantly lowered the MDA and LOX activities in strawberries, guava, avocado, and cherry during storage (Hong et al., 2012; Pasquariello et al., 2015; Petriccione et al., 2015; Obianom et al., 2019). Interestingly, chitosan-coated loquat fruit showed a relatively stable condition of LOX activity while the control fruit exhibited a sharp increase of this enzyme during storage (Song et al., 2016). The MDA content was also reduced by chitosan in the longan fruits during storage and in maize seedlings during growth, indicating an effective reduction of lipid peroxidation compared with the control (Qu et al., 2019; Lin et al., 2020). Chitosan forms a protective film on fruit surfaces upon treatment, which acts as a barrier to control O_2_ uptake, thus slowing respiration and delaying senescence (Romanazzi et al., 2017; Duan et al., 2019). Therefore, the chitosan films formed on fruit wounds upon treatment in this study slowed the senescence process and improved the mechanical strength of cell walls to prevent membrane breakdown compared to non-treated fruits.

Plant tissues undergo a mechanism that helps to maintain stable homeostasis (Zeng et al., 2010; Zhang et al., 2020). This mechanism depends on the levels of antioxidant enzymes (Rui et al., 2020). SOD, CAT, and POD are enzyme-based scavenging systems, while ascorbic acid, glutathione, and carotenoids are non-enzymatic scavenging systems (Gao et al., 2020). SOD removes ROS by reacting with O${}_{2}^{-}$ to form H_2_O_2_, while H_2_O_2_ breakdown is catalyzed by CAT, producing O_2_ and H_2_O in cell peroxisomes (Xue et al., 2019, 2020). H_2_O_2_ stimulates the POD activity in the AsA-GSH cycle to produce O_2_ and H_2_O during hydrolysis, and H_2_O_2_ is scavenged in this process (Wang and Gao, 2013; Han et al., 2021). From our results, the individual activities of SOD, CAT, and POD increased in all wounded fruits more than in non-wounded fruits due to wound stress. Meanwhile, the wounded fruits treated with chitosan had higher antioxidant activities than the control-wounded fruits (Figure 3). This result agrees with previous studies where chitosan increased the activities of CAT, GPX, and SOD in strawberries, cherries, and shoot cultures of lemon balm (Pasquariello et al., 2015; Petriccione et al., 2015; Fooladi vanda et al., 2019). Again, chitosan treatment increased SOD, CAT, POD, and PPO activities in fresh-cut apples and apricots (Cui et al., 2020; Zhao et al., 2021). According to Yang et al. (2020), increased H_2_O_2_ activates POD activity prior to lignin accumulation. Therefore, the increase in H_2_O_2_ content at the initial stage of this study induced the defense expression and helped activate POD, which can enhance structural barrier formation in fruit wounds. Chitosan's ability to remove ROS is mainly linked to its free amino ions and hydroxyl group, allowing the interactions of chitosan with negatively-charged molecules, that is, –NH_2_ can react with hydrogen atoms and further react with oxygen-like free radicals that are active to produce non-toxic and stable complexes (Duan et al., 2019; Hidangmayum et al., 2019). This complex process may scavenge excessive ROS. Xing et al. (2015) reported that chitosan induces resistance to stress by activating defense-related enzymes, such as phenylalanine ammonia-lyase, POD, PPO, CAT, and SOD, in fruits. Moreover, research shows that water-soluble chitosan has better preservative and antioxidant activity than acid-soluble chitosan due to differences in their physicochemical properties (Chung et al., 2011). Thus, we suggest that the water-soluble chitosan treatment used in our study is responsible for the significant increment in antioxidant enzyme activities to scavenge ROS in response to wounding stress.

The ascorbate-glutathione (AsA-GSH) cycle is an antioxidant pathway for the redox state of ascorbate and glutathione during stress (Shan et al., 2018). Ascorbate peroxidase (APX), monodehydroascorbate reductase (MDHAR), dehydroascorbate reductase (DHAR), and glutathione reductase (GR) are key enzymes regulating this pathway (Pasquariello et al., 2015; Rui et al., 2020). The APX aids in the conversion of H_2_O_2_ to H_2_O using AsA as the main electronic donor (Li et al., 2010b; Petriccione et al., 2015). Meanwhile, the activity of monodehydroascorbate reductase uses NADPH as an electron donor to dismutase MDHA into DHA or convert it to AsA (Rui et al., 2020). Then, DHAR converts DHA directly to AsA by using GSH. Conversely, with the aid of DHAR, GSH is also oxidized into GSSG, which is again reduced to GSH utilizing NADPH as the main electron donor by GR (Adiletta et al., 2021). According to our results, the activities of APX, MDHAR, DHAR, and GR, as well as AsA, DHA, GSH, and GSSG contents, were increased in wounded fruit than non-wounded fruit, however, wounded the fruit treated with chitosan showed higher activities compared to the control-wounded fruit (Figures 4, 5). Our results agree with that of Lin et al. (2020), who reported that chitosan increased the APX activity and AsA and GSH contents in longan fruits during storage. In addition, chitosan elicited the activities of MDHAR and DHAR, and increased the AsA and GSH contents in strawberry fruits to induce resistance and maintain quality (Wang and Gao, 2013). AsA in plant cells enables the breakdown of H_2_O_2_ free radicals into H_2_O under enhanced APX (Li et al., 2010b; Sharma et al., 2019), thus reducing ROS production. Glutathione is mainly in two forms: oxidized (GSSG) and reduced (GSH), however, the majority of glutathione is in the reduced form, which helps to maintain cellular redox balance (Shan et al., 2018). To reduce ROS, GSH reacts directly with many free radicals, like the hydroxyl radical (Wang and Gao, 2013). AsA and GSH control excessive ROS by either inhibiting oxidative chain reactions or dispelling reactive species (Zeng et al., 2010). The transcriptome data showed that chitosan significantly up-regulated genes associated with AsA and GSH in white clover to reduce ROS during drought (Li et al., 2017). Gu et al. (2021) reported that the up-regulation of LOC101295782 (APX) and ERD7 genes in strawberries by chitosan contributed to ROS reduction. Undoubtedly, the AsA-GSH cycle was elicited by chitosan in wounded fruit in this study, which facilitated the reduction of ROS and maintained the fruit membrane integrity, as shown in Figure 2. Thus, chitosan responds to wounding stress in fruit by the production of ROS to induce defense, and controls oxidative stress by eliciting antioxidant enzyme systems and pathways to prevent membrane damage.

## Conclusion

The use of chitosan in this study increased the activities of NOX and SOD for the production of O${}_{2}^{-}$ and H_2_O_2_ in fruit wounds. In addition, the stimulation of antioxidant systems caused ROS scavenging in fruit cells to reduce MDA, cell membrane permeability, and the activity of LOX. The activities of antioxidant enzymes, such as CAT and POD, as well as the enzymes associated with the ascorbate-glutathione cycle and the content of substrates and products at the fruit wounds, were increased by chitosan during storage. Therefore, chitosan proves to be more effective in regulating the formation and elimination of ROS to prevent oxidative stress and lipid peroxidation, thereby, maintaining post-harvest fruit quality under wound stress. This preservation strategy is an effective alternative and safer mean to extend the shelf life of various fruits during post-harvest storage.

## Data availability statement

The raw data supporting the conclusions of this article will be made available by the authors, without undue reservation, to any qualified researcher.

## Author contributions

SA and SX designed the experiment set up, performed the experiments, and analyzed data. SA wrote and edited the manuscript. SY, RA, and YZ analyzed data and revised manuscript. YH provided resources, supervision, and revised manuscript. YB and DP conceived the idea and revised the manuscript. YB designed the experiments, conceived the idea, funding, supervision, and revised the manuscript. All authors have read and agreed to the published version of the manuscript.

## Funding

This research work was financed by the China-Israel (NSFC-ISF) project of the National Natural Science Foundation of China (31861143046 and 31660476).

## Conflict of interest

The authors declare that the research was conducted in the absence of any commercial or financial relationships that could be construed as a potential conflict of interest.

## Publisher's note

All claims expressed in this article are solely those of the authors and do not necessarily represent those of their affiliated organizations, or those of the publisher, the editors and the reviewers. Any product that may be evaluated in this article, or claim that may be made by its manufacturer, is not guaranteed or endorsed by the publisher.
